# Supplementary material for: The metabolomic profile associated with clustering of cardiovascular risk factors—A multi-sample evaluation
Source: PLoS One. 2022 Sep 15;17(9):e0274701. doi: 10.1371/journal.pone.0274701 (PMC9477278; doi:10.1371/journal.pone.0274701)
Supplement: S1 Table — The criteria are given together with sum of criteria. The table was sorted on sum of criteria. (DOCX) [file pone.0274701.s001.docx]

**S1 Table. Overview of the metabolites being associated with at least one of the five MetS criteria.** The criteria are given together with sum of criteria. The table was sorted on sum of criteria.

| Chemical name | GLU | HDL | SBP | TG | WC | Sum of criteria |
| --- | --- | --- | --- | --- | --- | --- |
| N-formylphenylalanine | 0 | 0 | 1 | 0 | 0 | 1 |
| N1-Methyl-2-pyridone-5-carboxamide | 1 | 0 | 0 | 0 | 0 | 1 |
| arabinose | 1 | 0 | 0 | 0 | 0 | 1 |
| tyramine O-sulfate | 1 | 0 | 0 | 0 | 0 | 1 |
| 3-methoxytyramine sulfate | 0 | 0 | 0 | 1 | 0 | 1 |
| N6,N6-dimethyllysine | 1 | 0 | 0 | 0 | 0 | 1 |
| 7-methylguanine | 0 | 0 | 0 | 1 | 0 | 1 |
| glucuronate | 0 | 0 | 0 | 1 | 0 | 1 |
| N-acetylglutamine | 0 | 0 | 0 | 1 | 0 | 1 |
| tiglylcarnitine (C5:1-DC) | 0 | 1 | 0 | 0 | 0 | 1 |
| N-palmitoylglycine | 0 | 1 | 0 | 0 | 0 | 1 |
| N6-succinyladenosine | 0 | 0 | 0 | 1 | 0 | 1 |
| glycolithocholate | 0 | 0 | 0 | 1 | 0 | 1 |
| androstenediol (3alpha, 17alpha) monosulfate (2) | 0 | 1 | 0 | 0 | 0 | 1 |
| phenol glucuronide | 0 | 0 | 0 | 1 | 0 | 1 |
| 5alpha-androstan-3beta,17alpha-diol disulfate | 0 | 0 | 0 | 1 | 0 | 1 |
| cytidine | 0 | 1 | 0 | 0 | 0 | 1 |
| hexanoylcarnitine (C6) | 0 | 1 | 0 | 0 | 0 | 1 |
| pentadecanoate (15:0) | 0 | 1 | 0 | 0 | 0 | 1 |
| glycoursodeoxycholate | 0 | 0 | 0 | 1 | 0 | 1 |
| anthranilate | 1 | 0 | 0 | 0 | 0 | 1 |
| hydroxy-N6,N6,N6-trimethyllysine | 0 | 1 | 0 | 0 | 0 | 1 |
| 2-hydroxyglutarate | 0 | 1 | 0 | 0 | 0 | 1 |
| vanillylmandelate (VMA) | 0 | 0 | 1 | 0 | 0 | 1 |
| vanillactate | 0 | 0 | 0 | 1 | 0 | 1 |
| phenylacetate | 0 | 0 | 0 | 1 | 0 | 1 |
| chiro-inositol | 0 | 1 | 0 | 0 | 0 | 1 |
| 4-hydroxy-2-oxoglutaric acid | 0 | 0 | 0 | 1 | 0 | 1 |
| cis-4-decenoylcarnitine (C10:1) | 1 | 0 | 0 | 0 | 0 | 1 |
| arabitol/xylitol | 0 | 0 | 0 | 1 | 0 | 1 |
| eicosenedioate (C20:1-DC) | 0 | 0 | 0 | 1 | 0 | 1 |
| 2-hydroxy-4-(methylthio)butanoic acid | 1 | 0 | 0 | 0 | 0 | 1 |
| threonine | 0 | 0 | 0 | 1 | 0 | 1 |
| dihomo-linolenoyl-choline | 1 | 0 | 0 | 0 | 0 | 1 |
| methylsuccinoylcarnitine | 1 | 0 | 0 | 0 | 0 | 1 |
| cholic acid glucuronide | 1 | 0 | 0 | 0 | 0 | 1 |
| leucylglycine | 1 | 0 | 0 | 0 | 0 | 1 |
| N,N,N-trimethyl-alanylproline betaine (TMAP) | 1 | 0 | 0 | 0 | 0 | 1 |
| carnitine | 0 | 0 | 0 | 1 | 0 | 1 |
| glycerol | 0 | 1 | 0 | 0 | 0 | 1 |
| androsterone sulfate | 0 | 1 | 0 | 0 | 0 | 1 |
| trimethylamine N-oxide | 1 | 0 | 0 | 0 | 0 | 1 |
| 2S,3R-dihydroxybutyrate | 0 | 1 | 0 | 0 | 0 | 1 |
| N-acetyltaurine | 1 | 0 | 0 | 0 | 0 | 1 |
| xanthine | 0 | 0 | 0 | 1 | 0 | 1 |
| riboflavin (Vitamin B2) | 0 | 0 | 0 | 0 | 1 | 1 |
| beta-hydroxyisovaleroylcarnitine | 0 | 1 | 0 | 0 | 0 | 1 |
| glycocholate | 0 | 0 | 0 | 1 | 0 | 1 |
| epiandrosterone sulfate | 0 | 0 | 0 | 1 | 0 | 1 |
| 5-hydroxyhexanoate | 0 | 1 | 0 | 0 | 0 | 1 |
| butyrate/isobutyrate (4:0) | 1 | 0 | 0 | 0 | 0 | 1 |
| 4-methoxyphenol sulfate | 1 | 0 | 0 | 0 | 0 | 1 |
| glutarylcarnitine (C5-DC) | 1 | 0 | 0 | 0 | 0 | 1 |
| kynurenine | 0 | 1 | 0 | 0 | 0 | 1 |
| linoleate (18:2n6) | 0 | 1 | 0 | 0 | 0 | 1 |
| inosine | 1 | 0 | 0 | 0 | 0 | 1 |
| taurodeoxycholic acid 3-sulfate | 1 | 0 | 0 | 0 | 0 | 1 |
| xylose | 0 | 0 | 0 | 1 | 0 | 1 |
| glycochenodeoxycholate | 0 | 0 | 0 | 1 | 0 | 1 |
| tauroursodeoxycholate | 0 | 0 | 0 | 1 | 0 | 1 |
| carnosine | 0 | 0 | 0 | 1 | 0 | 1 |
| hydantoin-5-propionate | 0 | 1 | 0 | 0 | 0 | 1 |
| cis-4-decenoate (10:1n6) | 0 | 1 | 0 | 0 | 0 | 1 |
| 5alpha-pregnan-3beta,20alpha-diol monosulfate (2) | 0 | 0 | 0 | 1 | 0 | 1 |
| indolelactate | 0 | 0 | 0 | 1 | 0 | 1 |
| lanthionine | 0 | 0 | 0 | 1 | 0 | 1 |
| 3-hydroxyoleoylcarnitine | 0 | 1 | 0 | 0 | 0 | 1 |
| cysteinylglycine | 0 | 0 | 0 | 1 | 0 | 1 |
| 3-amino-2-piperidone | 0 | 0 | 0 | 1 | 0 | 1 |
| p-cresol glucuronide | 0 | 0 | 0 | 0 | 1 | 1 |
| lysine | 0 | 1 | 0 | 0 | 0 | 1 |
| caproate (6:0) | 0 | 1 | 0 | 0 | 0 | 1 |
| dimethylglycine | 0 | 1 | 0 | 0 | 0 | 1 |
| 2-hydroxystearate | 0 | 1 | 0 | 0 | 0 | 1 |
| cysteine s-sulfate | 0 | 0 | 0 | 1 | 0 | 1 |
| taurochenodeoxycholate | 0 | 0 | 1 | 0 | 0 | 1 |
| N-acetylglucosaminylasparagine | 0 | 0 | 0 | 1 | 0 | 1 |
| 2-hydroxysebacate | 0 | 1 | 0 | 0 | 0 | 1 |
| pro-hydroxy-pro | 1 | 0 | 0 | 0 | 0 | 1 |
| N-acetylmethionine | 0 | 1 | 0 | 0 | 0 | 1 |
| phenyllactate (PLA) | 0 | 1 | 0 | 0 | 0 | 1 |
| 3beta,7alpha-dihydroxy-5-cholestenoate | 0 | 0 | 0 | 1 | 0 | 1 |
| 3b-hydroxy-5-cholenoic acid | 0 | 1 | 0 | 0 | 0 | 1 |
| acisoga | 0 | 0 | 0 | 1 | 0 | 1 |
| azelate (C9-DC) | 1 | 0 | 0 | 0 | 0 | 1 |
| 5alpha-androstan-3alpha,17alpha-diol monosulfate | 0 | 0 | 0 | 1 | 0 | 1 |
| gamma-CEHC | 0 | 0 | 0 | 1 | 0 | 1 |
| gamma-glutamyl-alpha-lysine | 0 | 1 | 0 | 0 | 0 | 1 |
| branched-chain, straight-chain, or cyclopropyl 10:1 fatty acid (3)* | 0 | 1 | 0 | 0 | 0 | 1 |
| alpha-ketobutyrate | 1 | 0 | 0 | 0 | 0 | 1 |
| N-oleoyltaurine | 0 | 1 | 0 | 0 | 0 | 1 |
| succinoyltaurine | 0 | 0 | 0 | 1 | 0 | 1 |
| taurolithocholate 3-sulfate | 0 | 0 | 0 | 1 | 0 | 1 |
| 1-linoleoyl-GPG (18:2) | 0 | 0 | 0 | 1 | 0 | 1 |
| allantoin | 1 | 0 | 0 | 0 | 0 | 1 |
| homovanillate (HVA) | 0 | 0 | 0 | 1 | 0 | 1 |
| (14 or 15)-methylpalmitate (a17:0 or i17:0) | 0 | 1 | 0 | 0 | 0 | 1 |
| phenol sulfate | 0 | 0 | 0 | 1 | 0 | 1 |
| isovalerylglycine | 0 | 0 | 1 | 0 | 0 | 1 |
| uridine | 0 | 1 | 0 | 0 | 0 | 1 |
| 3-hydroxystearate | 0 | 1 | 0 | 0 | 0 | 1 |
| linoleoyl ethanolamide | 0 | 1 | 0 | 0 | 0 | 1 |
| 3-(4-hydroxyphenyl)lactate | 0 | 0 | 0 | 1 | 0 | 1 |
| phosphate | 0 | 1 | 0 | 0 | 0 | 1 |
| nicotinamide riboside | 0 | 1 | 0 | 0 | 0 | 1 |
| 1-methyl-5-imidazoleacetate | 0 | 0 | 0 | 1 | 0 | 1 |
| adenosine 5'-monophosphate (AMP) | 1 | 0 | 0 | 0 | 0 | 1 |
| retinal | 0 | 0 | 0 | 1 | 0 | 1 |
| etiocholanolone glucuronide | 0 | 1 | 0 | 0 | 0 | 1 |
| N-acetyl-2-aminoadipate | 0 | 0 | 0 | 0 | 1 | 1 |
| citraconate/glutaconate | 0 | 1 | 0 | 0 | 0 | 1 |
| N6-carboxymethyllysine | 1 | 0 | 0 | 0 | 0 | 1 |
| homocitrulline | 0 | 0 | 0 | 1 | 0 | 1 |
| chenodeoxycholate | 0 | 1 | 0 | 0 | 0 | 1 |
| malonylcarnitine | 0 | 0 | 0 | 1 | 0 | 1 |
| pantoate | 0 | 0 | 0 | 0 | 1 | 1 |
| sphingadienine | 0 | 1 | 0 | 0 | 0 | 1 |
| S-1-pyrroline-5-carboxylate | 1 | 0 | 0 | 0 | 0 | 1 |
| 3-methylglutaconate | 1 | 0 | 0 | 0 | 0 | 1 |
| glycerol 3-phosphate | 0 | 0 | 0 | 1 | 0 | 1 |
| alpha-CEHC sulfate | 0 | 0 | 0 | 1 | 0 | 1 |
| uracil | 1 | 0 | 0 | 0 | 0 | 1 |
| histidine | 1 | 0 | 0 | 0 | 0 | 1 |
| N-palmitoylserine | 0 | 0 | 0 | 1 | 0 | 1 |
| 2,3-dihydroxy-2-methylbutyrate | 0 | 0 | 0 | 1 | 0 | 1 |
| heptanoate (7:0) | 0 | 1 | 0 | 0 | 0 | 1 |
| nicotinamide | 1 | 0 | 0 | 0 | 0 | 1 |
| 5alpha-androstan-3alpha,17beta-diol disulfate | 1 | 0 | 0 | 0 | 0 | 1 |
| methylsuccinate | 0 | 0 | 0 | 1 | 0 | 1 |
| 1-oleoyl-2-docosahexaenoyl-GPE (18:1/22:6) | 0 | 0 | 0 | 1 | 0 | 1 |
| phenylacetylglutamine | 0 | 0 | 0 | 1 | 0 | 1 |
| gamma-glutamyltryptophan | 0 | 0 | 0 | 1 | 0 | 1 |
| prolylglycine | 0 | 0 | 0 | 1 | 0 | 1 |
| N-acetyl-1-methylhistidine | 0 | 0 | 0 | 1 | 0 | 1 |
| octadecanedioylcarnitine (C18-DC) | 0 | 0 | 0 | 1 | 0 | 1 |
| thyroxine | 0 | 1 | 0 | 0 | 0 | 1 |
| glycodeoxycholate 3-sulfate | 1 | 0 | 0 | 0 | 0 | 1 |
| glutamine_degradant | 1 | 0 | 0 | 0 | 0 | 1 |
| arabonate/xylonate | 0 | 0 | 0 | 1 | 0 | 1 |
| N6-acetyllysine | 1 | 0 | 0 | 0 | 0 | 1 |
| N-acetyltryptophan | 0 | 0 | 0 | 1 | 0 | 1 |
| pregnanediol-3-glucuronide | 0 | 0 | 0 | 1 | 0 | 1 |
| isobutyrylglycine | 0 | 0 | 1 | 0 | 0 | 1 |
| glutarate (C5-DC) | 0 | 0 | 0 | 1 | 0 | 1 |
| carboxyethyl-GABA | 0 | 1 | 0 | 0 | 0 | 1 |
| methionine sulfoxide | 0 | 0 | 0 | 0 | 1 | 1 |
| dehydroepiandrosterone sulfate (DHEA-S) | 1 | 0 | 0 | 0 | 0 | 1 |
| andro steroid monosulfate C19H28O6S (1) | 0 | 0 | 1 | 0 | 0 | 1 |
| hyocholate | 0 | 0 | 0 | 1 | 0 | 1 |
| adipoylcarnitine (C6-DC) | 0 | 0 | 0 | 1 | 0 | 1 |
| 1,5-anhydroglucitol (1,5-AG) | 1 | 0 | 0 | 0 | 0 | 1 |
| sphingomyelin (d18:1/24:1, d18:2/24:0) | 1 | 1 | 0 | 0 | 0 | 2 |
| N2,N5-diacetylornithine | 0 | 1 | 0 | 0 | 1 | 2 |
| 4-acetamidobutanoate | 0 | 1 | 0 | 1 | 0 | 2 |
| N,N-dimethyl-pro-pro | 1 | 1 | 0 | 0 | 0 | 2 |
| urea | 0 | 0 | 1 | 1 | 0 | 2 |
| nonanoylcarnitine (C9) | 1 | 1 | 0 | 0 | 0 | 2 |
| tetradecanedioate (C14-DC) | 0 | 1 | 1 | 0 | 0 | 2 |
| linolenoylcarnitine (C18:3) | 1 | 1 | 0 | 0 | 0 | 2 |
| 1-linoleoylglycerol (18:2) | 0 | 1 | 0 | 1 | 0 | 2 |
| 1-methyl-4-imidazoleacetate | 0 | 1 | 0 | 1 | 0 | 2 |
| N-acetylcarnosine | 0 | 1 | 0 | 1 | 0 | 2 |
| undecenoylcarnitine (C11:1) | 1 | 1 | 0 | 0 | 0 | 2 |
| N,N-dimethylalanine | 1 | 0 | 0 | 1 | 0 | 2 |
| N-delta-acetylornithine | 0 | 1 | 0 | 0 | 1 | 2 |
| taurocholenate sulfate | 1 | 0 | 0 | 1 | 0 | 2 |
| N6,N6,N6-trimethyllysine | 1 | 1 | 0 | 0 | 0 | 2 |
| homoarginine | 0 | 1 | 0 | 1 | 0 | 2 |
| 21-hydroxypregnenolone disulfate | 1 | 0 | 0 | 1 | 0 | 2 |
| biliverdin | 0 | 1 | 0 | 1 | 0 | 2 |
| 6-oxopiperidine-2-carboxylate | 1 | 1 | 0 | 0 | 0 | 2 |
| 3-methoxytyrosine | 1 | 0 | 0 | 1 | 0 | 2 |
| pregnenediol sulfate (C21H34O5S) | 1 | 0 | 0 | 1 | 0 | 2 |
| tyrosine | 0 | 1 | 0 | 1 | 0 | 2 |
| 2-hydroxybutyrate/2-hydroxyisobutyrate | 1 | 1 | 0 | 0 | 0 | 2 |
| 1-(1-enyl-stearoyl)-2-arachidonoyl-GPE (P-18:0/20:4) | 0 | 1 | 0 | 1 | 0 | 2 |
| 2-hydroxyphenylacetate | 0 | 1 | 0 | 1 | 0 | 2 |
| decanoylcarnitine (C10) | 1 | 1 | 0 | 0 | 0 | 2 |
| sphingomyelin (d18:1/19:0, d19:1/18:0) | 1 | 1 | 0 | 0 | 0 | 2 |
| 2-hydroxy-3-methylvalerate | 1 | 0 | 0 | 1 | 0 | 2 |
| malate | 1 | 1 | 0 | 0 | 0 | 2 |
| alpha-hydroxyisocaproate | 1 | 0 | 0 | 1 | 0 | 2 |
| branched-chain, straight-chain, or cyclopropyl 12:1 fatty acid | 0 | 1 | 1 | 0 | 0 | 2 |
| aconitate [cis or trans] | 1 | 0 | 0 | 1 | 0 | 2 |
| behenoylcarnitine (C22) | 0 | 1 | 0 | 1 | 0 | 2 |
| dimethylarginine (SDMA + ADMA) | 1 | 1 | 0 | 0 | 0 | 2 |
| 2-hydroxypalmitate | 0 | 1 | 0 | 0 | 1 | 2 |
| taurine | 1 | 1 | 0 | 0 | 0 | 2 |
| phenylacetylcarnitine | 0 | 1 | 1 | 0 | 0 | 2 |
| 3-ureidopropionate | 1 | 0 | 0 | 1 | 0 | 2 |
| isobutyrylcarnitine (C4) | 0 | 1 | 1 | 0 | 0 | 2 |
| xanthosine | 0 | 1 | 0 | 1 | 0 | 2 |
| glycochenodeoxycholate glucuronide (1) | 1 | 1 | 0 | 0 | 0 | 2 |
| lyxonate | 1 | 1 | 0 | 0 | 0 | 2 |
| 3-methyl-2-oxobutyrate | 1 | 0 | 0 | 1 | 0 | 2 |
| bilirubin degradation product, C16H18N2O5 (1) | 0 | 1 | 0 | 1 | 0 | 2 |
| gamma-glutamylalanine | 1 | 0 | 0 | 1 | 0 | 2 |
| acetylcarnitine (C2) | 0 | 1 | 0 | 1 | 0 | 2 |
| 3-hydroxyhexanoylcarnitine (1) | 1 | 0 | 0 | 1 | 0 | 2 |
| branched chain 14:0 dicarboxylic acid | 0 | 0 | 0 | 1 | 1 | 2 |
| alpha-ketoglutarate | 1 | 0 | 0 | 1 | 0 | 2 |
