# Supplementary material for: The metabolomic profile associated with clustering of cardiovascular risk factors—A multi-sample evaluation
Source: PLoS One. 2022 Sep 15;17(9):e0274701. doi: 10.1371/journal.pone.0274701 (PMC9477278; doi:10.1371/journal.pone.0274701)
Supplement: S2 Table — The estimates are from the validation step in the SCAPIS-Malmö cohort. No relationship for the metabolite glucose vs the GLU criteria is shown. (DOCX) [file pone.0274701.s002.docx]

**S2 Table. Relationships between the 15 validated metabolites being related to all five components in the metabolic syndrome (MetS) and fasting glucose (GLU), HDL-cholesterol, systolic blood pressure (SBP), triglycerides (TG) and waist circumference (WC).** The estimates are from the validation step in the SCAPIS-Malmö cohort. No relationship for the metabolite glucose vs the GLU criteria is shown.

| Super pathway | Sub pathway | Chemical name | MetS criteria | Beta | 95%CI lower | 95%CI higher | p-value |
| --- | --- | --- | --- | --- | --- | --- | --- |
| Amino Acid | Glycine, Serine and Threonine Metabolism | glycine | GLU | -.10 | -.13 | -.07 | 5.72e-09 |
| Amino Acid | Glycine, Serine and Threonine Metabolism | glycine | HDL | .05 | .03 | .07 | .00017 |
| Amino Acid | Glycine, Serine and Threonine Metabolism | glycine | SBP | -.05 | -.09 | -.01 | .0060 |
| Amino Acid | Glycine, Serine and Threonine Metabolism | glycine | TG | -.16 | -.19 | -.13 | 9.00e-24 |
| Amino Acid | Glycine, Serine and Threonine Metabolism | glycine | WC | -.05 | -.06 | -.04 | 4.14e-10 |
| Amino Acid | Methionine, Cysteine, SAM and Taurine Metabolism | S-methylcysteine sulfoxide | GLU | -.06 | -.09 | -.03 | .00036 |
| Amino Acid | Methionine, Cysteine, SAM and Taurine Metabolism | S-methylcysteine sulfoxide | HDL | .04 | .02 | .06 | .0017 |
| Amino Acid | Methionine, Cysteine, SAM and Taurine Metabolism | S-methylcysteine sulfoxide | SBP | -.07 | -.10 | -.04 | .000075 |
| Amino Acid | Methionine, Cysteine, SAM and Taurine Metabolism | S-methylcysteine sulfoxide | TG | -.11 | -.14 | -.08 | 1.41e-13 |
| Amino Acid | Methionine, Cysteine, SAM and Taurine Metabolism | S-methylcysteine sulfoxide | WC | -.03 | -.04 | -.02 | .000030 |
| Carbohydrate | Glycolysis, Gluconeogenesis, and Pyruvate Metabolism | glucose | GLU | - | - | - | - |
| Carbohydrate | Glycolysis, Gluconeogenesis, and Pyruvate Metabolism | glucose | HDL | -.09 | -.12 | -.06 | 4.28e-11 |
| Carbohydrate | Glycolysis, Gluconeogenesis, and Pyruvate Metabolism | glucose | SBP | .06 | .02 | .1 | .00087 |
| Carbohydrate | Glycolysis, Gluconeogenesis, and Pyruvate Metabolism | glucose | TG | .16 | .13 | .19 | 1.00e-24 |
| Carbohydrate | Glycolysis, Gluconeogenesis, and Pyruvate Metabolism | glucose | WC | .05 | .04 | .06 | 5.55e-13 |
| Carbohydrate | Glycolysis, Gluconeogenesis, and Pyruvate Metabolism | glycerate | GLU | -.05 | -.09 | -.01 | .00085 |
| Carbohydrate | Glycolysis, Gluconeogenesis, and Pyruvate Metabolism | glycerate | HDL | .1 | .07 | .13 | 2.57e-12 |
| Carbohydrate | Glycolysis, Gluconeogenesis, and Pyruvate Metabolism | glycerate | SBP | -.06 | -.1 | -.02 | .00035 |
| Carbohydrate | Glycolysis, Gluconeogenesis, and Pyruvate Metabolism | glycerate | TG | -.12 | -.15 | -.09 | 2.49e-16 |
| Carbohydrate | Glycolysis, Gluconeogenesis, and Pyruvate Metabolism | glycerate | WC | -.05 | -.06 | -.04 | 3.60e-11 |
| Carbohydrate | Glycolysis, Gluconeogenesis, and Pyruvate Metabolism | lactate | GLU | .14 | .11 | .17 | 3.11e-19 |
| Carbohydrate | Glycolysis, Gluconeogenesis, and Pyruvate Metabolism | lactate | HDL | -.08 | -.1 | -.06 | 1.63e-08 |
| Carbohydrate | Glycolysis, Gluconeogenesis, and Pyruvate Metabolism | lactate | SBP | .06 | .02 | .1 | .00074 |
| Carbohydrate | Glycolysis, Gluconeogenesis, and Pyruvate Metabolism | lactate | TG | .28 | .25 | .31 | <1.00e-25 |
| Carbohydrate | Glycolysis, Gluconeogenesis, and Pyruvate Metabolism | lactate | WC | .03 | .02 | .04 | 5.44e-07 |
| Cofactors and Vitamins | Ascorbate and Aldarate Metabolism | oxalate (ethanedioate) | GLU | -.03 | -.06 | 0 | .076 |
| Cofactors and Vitamins | Ascorbate and Aldarate Metabolism | oxalate (ethanedioate) | HDL | .09 | .06 | .12 | 8.40e-10 |
| Cofactors and Vitamins | Ascorbate and Aldarate Metabolism | oxalate (ethanedioate) | SBP | -.08 | -.11 | -.05 | .000026 |
| Cofactors and Vitamins | Ascorbate and Aldarate Metabolism | oxalate (ethanedioate) | TG | -.12 | -.15 | -.09 | 1.28e-13 |
| Cofactors and Vitamins | Ascorbate and Aldarate Metabolism | oxalate (ethanedioate) | WC | -.05 | -.06 | -.04 | 6.60e-11 |
| Cofactors and Vitamins | Vitamin A Metabolism | carotene diol (2) | GLU | -.07 | -.1 | -.04 | .000059 |
| Cofactors and Vitamins | Vitamin A Metabolism | carotene diol (2) | HDL | .16 | .13 | .19 | <1.00e-25 |
| Cofactors and Vitamins | Vitamin A Metabolism | carotene diol (2) | SBP | -.06 | -.09 | -.03 | .0021 |
| Cofactors and Vitamins | Vitamin A Metabolism | carotene diol (2) | TG | -.18 | -.21 | -.15 | <1.00e-25 |
| Cofactors and Vitamins | Vitamin A Metabolism | carotene diol (2) | WC | -.07 | -.09 | -.05 | 8.00e-24 |
| Lipid | Androgenic Steroids | 11beta-hydroxyandrosterone glucuronide | GLU | .08 | .05 | .11 | 2.65e-07 |
| Lipid | Androgenic Steroids | 11beta-hydroxyandrosterone glucuronide | HDL | -.1 | -.12 | -.08 | 1.25e-12 |
| Lipid | Androgenic Steroids | 11beta-hydroxyandrosterone glucuronide | SBP | .05 | .02 | .08 | .0022 |
| Lipid | Androgenic Steroids | 11beta-hydroxyandrosterone glucuronide | TG | .09 | .06 | .12 | 1.15e-08 |
| Lipid | Androgenic Steroids | 11beta-hydroxyandrosterone glucuronide | WC | .05 | .03 | .07 | 3.24e-11 |
| Lipid | Corticosteroids | cortolone glucuronide (1) | GLU | .06 | .02 | .1 | .00092 |
| Lipid | Corticosteroids | cortolone glucuronide (1) | HDL | -.04 | -.07 | -.01 | .01011 |
| Lipid | Corticosteroids | cortolone glucuronide (1) | SBP | .07 | .03 | .11 | .00023 |
| Lipid | Corticosteroids | cortolone glucuronide (1) | TG | .11 | .08 | .14 | 6.54e-12 |
| Lipid | Corticosteroids | cortolone glucuronide (1) | WC | .07 | .05 | .09 | 2.20e-20 |
| Lipid | Corticosteroids | tetrahydrocortisol glucuronide | GLU | .13 | .09 | .17 | 4.69e-14 |
| Lipid | Corticosteroids | tetrahydrocortisol glucuronide | HDL | -.11 | -.13 | -.09 | 7.39e-14 |
| Lipid | Corticosteroids | tetrahydrocortisol glucuronide | SBP | .07 | .03 | .11 | .00035 |
| Lipid | Corticosteroids | tetrahydrocortisol glucuronide | TG | .19 | .16 | .22 | <1.00e-25 |
| Lipid | Corticosteroids | tetrahydrocortisol glucuronide | WC | .05 | .04 | .06 | 1.73e-12 |
| Lipid | Dihydroceramides | N-stearoyl-sphinganine (d18:0/18:0)* | GLU | .08 | .04 | .12 | .00013 |
| Lipid | Dihydroceramides | N-stearoyl-sphinganine (d18:0/18:0)* | HDL | -.1 | -.13 | -.07 | 2.14e-09 |
| Lipid | Dihydroceramides | N-stearoyl-sphinganine (d18:0/18:0)* | SBP | .07 | .03 | .11 | .0015 |
| Lipid | Dihydroceramides | N-stearoyl-sphinganine (d18:0/18:0)* | TG | .31 | .28 | .34 | <1.00e-25 |
| Lipid | Dihydroceramides | N-stearoyl-sphinganine (d18:0/18:0)* | WC | .08 | .06 | .1 | 3.71e-18 |
| Lipid | Fatty Acid Metabolism (Acyl Carnitine, Dicarboxylate) | pimeloylcarnitine/3-methyladipoylcarnitine (C7-DC) | GLU | -.11 | -.15 | -.07 | 6.40e-13 |
| Lipid | Fatty Acid Metabolism (Acyl Carnitine, Dicarboxylate) | pimeloylcarnitine/3-methyladipoylcarnitine (C7-DC) | HDL | .08 | .05 | .11 | 1.36e-08 |
| Lipid | Fatty Acid Metabolism (Acyl Carnitine, Dicarboxylate) | pimeloylcarnitine/3-methyladipoylcarnitine (C7-DC) | SBP | -.05 | -.08 | -.02 | .0047 |
| Lipid | Fatty Acid Metabolism (Acyl Carnitine, Dicarboxylate) | pimeloylcarnitine/3-methyladipoylcarnitine (C7-DC) | TG | -.16 | -.19 | -.13 | <1.00e-25 |
| Lipid | Fatty Acid Metabolism (Acyl Carnitine, Dicarboxylate) | pimeloylcarnitine/3-methyladipoylcarnitine (C7-DC) | WC | -.02 | -.03 | -.01 | .0030 |
| Lipid | Fatty Acid, Dicarboxylate | hydroxy-CMPF* | GLU | -.07 | -.1 | -.04 | .000049 |
| Lipid | Fatty Acid, Dicarboxylate | hydroxy-CMPF* | HDL | .1 | .08 | .12 | 1.06e-13 |
| Lipid | Fatty Acid, Dicarboxylate | hydroxy-CMPF* | SBP | -.05 | -.08 | -.02 | .0066 |
| Lipid | Fatty Acid, Dicarboxylate | hydroxy-CMPF* | TG | -.15 | -.18 | -.12 | 9.30e-23 |
| Lipid | Fatty Acid, Dicarboxylate | hydroxy-CMPF* | WC | -.02 | -.03 | -.01 | .0081 |
| Lipid | Phosphatidylethanolamine (PE) | 1-palmitoyl-2-oleoyl-GPE (16:0/18:1) | GLU | .03 | 0 | .06 | .072 |
| Lipid | Phosphatidylethanolamine (PE) | 1-palmitoyl-2-oleoyl-GPE (16:0/18:1) | HDL | -.14 | -.17 | -.11 | <1.00e-25 |
| Lipid | Phosphatidylethanolamine (PE) | 1-palmitoyl-2-oleoyl-GPE (16:0/18:1) | SBP | .07 | .04 | .1 | .0000263 |
| Lipid | Phosphatidylethanolamine (PE) | 1-palmitoyl-2-oleoyl-GPE (16:0/18:1) | TG | .58 | .55 | .61 | <1.00e-25 |
| Lipid | Phosphatidylethanolamine (PE) | 1-palmitoyl-2-oleoyl-GPE (16:0/18:1) | WC | .04 | .03 | .05 | 1.23e-08 |
| Partially Characterized Molecules | Partially Characterized Molecules | metabolonic lactone sulfate | GLU | .08 | .05 | .11 | 1.55e-06 |
| Partially Characterized Molecules | Partially Characterized Molecules | metabolonic lactone sulfate | HDL | -.2 | -.23 | -.17 | <1.00e-25 |
| Partially Characterized Molecules | Partially Characterized Molecules | metabolonic lactone sulfate | SBP | .06 | .02 | .1 | .0036 |
| Partially Characterized Molecules | Partially Characterized Molecules | metabolonic lactone sulfate | TG | .24 | .2 | .28 | <1.00e-25 |
| Partially Characterized Molecules | Partially Characterized Molecules | metabolonic lactone sulfate | WC | .05 | .04 | .06 | 7.47e-12 |
